# Discovery of a new konkovirus species in Lachenalia plants reveals possible co-evolution between 5′ and 3′ RNA sequence motifs

**DOI:** 10.1099/jgv.0.002159

**Published:** 2025-10-08

**Authors:** Rob J. Dekker, Wim C. de Leeuw, Marina F. van Olst, Wim A. Ensink, Selina M. van Leeuwen, Timo M. Breit, Martijs J. Jonker

**Affiliations:** 1 RNA Biology research group, Swammerdam Institute for Life Sciences, Faculty of Science, University of Amsterdam, Amsterdam, the Netherlands

**Keywords:** *Konkoviridae*, *Lachenalia*, plant viruses, RNA-dependent RNA polymerase (RdRp), RNA viruses, virus discovery

## Abstract

This study reports the discovery of a new konkovirus species, named *Lachenalia konkovirus* 1 (LaKoV1), from *Lachenalia* plants in an urban botanic garden in Amsterdam. Using a combination of RNA sequencing (RNA-seq), small RNA-seq and advanced bioinformatics, we identified a segmented, negative-strand RNA virus belonging to the family *Konkoviridae*. Our findings show significant divergence between this novel virus and known members of the family *Konkoviridae*, such as tulip streak virus (TuSV) and *Lactuca* big vein-associated *Phlebovirus* (LBVaPV), supporting its classification as a distinct species. Notably, the sequence differences found in the conserved 5′ and 3′ ends of these segments suggest potential co-evolution. Despite the observed genomic distances, there is significant conservation in the RNA-dependent RNA polymerase subdomain, underscoring evolutionary relationships among LaKoV1, TuSV and LBVaPV. Our findings expand the known global virome and highlight the importance of exploring plant viromes in diverse ecological settings to better understand virus evolution and diversity.

## Introduction

The global virome still remains largely unknown [1,2]. As a first step to understand the innumerable strategies viruses possess to evade the host anti-virus responses, it is important to discover as many different viruses as possible. This applies to human-pathogenic viruses [3,4], as well as to phytopathogenic viruses, which are a serious threat to global food security [5,6]. A particular environment that might be a breeding ground for, yet unknown, phytoviruses is the (urban) botanic garden. Given the green ‘islands’ that botanic gardens represent, where plants are kept in relative seclusion for numerous generations, often in close proximity to exotic plant species from exotic places, the assumption is that there may be many (yet unknown) viruses present in botanic garden plants.

There are several ways to discover new phytoviruses. The most straightforward approach is to analyse plant RNA with RNA sequencing (RNA-seq) and use bioinformatics to assemble the reads into contigs. The genome sequence of these contigs, or their translated protein sequences, can be compared to virus databases to detect known or yet-unknown virus sequences. One problem with the RNA-seq approach is the massive background of host RNA. Also, past virus infections cannot be detected by the lack of viral RNA. Another approach is to investigate the virus-related small interfering RNA (siRNA) defence response of the host plants to a viral infection [7]. Given the massive siRNA response of most plants to virus infection, which usually covers the entire length of RNA viruses, it is often possible to reconstruct the complete virus RNA sequence from the host-generated siRNAs [8]. A disadvantage of the siRNA approach relates to virus infections with multiple variants. As the typical length of siRNAs is 21 nucleotides, it is often difficult to entangle multiple virus variant sequences. This is obviously a lesser problem with paired-end 150-nucleotide RNA-seq reads. Hence, combining small RNA-seq and RNA-seq in virus discovery experiments will provide the best of both approaches.

To investigate the current or past presence of known virus variants or unknown viruses in botanic garden plants, we screened 25 *Asparagales* samples from a Dutch urban botanic garden by high-throughput small RNA-seq, as well as RNA-seq. In this report, we describe the discovery of a novel segmented, negative-sense RNA bunyavirus that appears to belong to the *Konkoviridae* family. The sequence differences found in the conserved 5′ and 3′ ends of the four virus RNA segments hint at the possible presence of sequence co-evolution. This new virus was discovered along with two other newly identified viruses within the same sample set [9,10].

## Methods

### Samples

Samples of leaves from 25 *Asparagales* plants were collected from Hortus Botanicus, a botanic garden in Amsterdam, the Netherlands, on 14 February 2019. Details of the plant genera can be found in Table ST1, available in the online Supplementary Material.

### RNA isolation

Small RNA was isolated by grinding a flash-frozen ±1 cm^2^ leaf fragment into fine powder using a mortar and pestle, dissolving the powder in QIAzol Lysis Reagent (Qiagen) and purifying the RNA using the miRNeasy Mini Kit (Qiagen). Separation of total RNA into small (<200 nt) and large (>200 nt) fractions, including DNase treatment of the large RNA isolates, was performed as described in the manufacturer’s instructions. The concentration of the RNA was determined using a NanoDrop ND-2000 (Thermo Fisher Scientific), and RNA quality was assessed using the 2200 TapeStation System Agilent RNA ScreenTapes (Agilent Technologies).

### RNA-seq

Barcoded small RNA-seq and RNA-seq libraries were generated using a Small RNA-Seq Library Prep Kit (Lexogen) and a TruSeq Stranded Total RNA with Ribo-Zero Plant Kit (Illumina), respectively. The size distribution of libraries with indexed adapters was assessed using a 2200 TapeStation System with Agilent D1000 ScreenTape (Agilent Technologies). The small RNA-seq libraries from samples S01–S12 and S14–S26 were clustered and sequenced at 2×75 and 1×75 bp, on a NextSeq 550 System using either a NextSeq 500/550 Mid Output Kit v2.5 or a NextSeq 500/550 High Output Kit v2.5 (75 cycles or 150 cycles; Illumina), respectively. RNA-seq libraries were clustered and sequenced at 2×150 bp on a NovaSeq 6000 System using the NovaSeq 6000 S4 Reagent Kit v1.5 (300 cycles; Illumina).

### Bioinformatics analyses

Sequencing reads were trimmed using trimmomatic v0.39 [11] (parameters: LEADING:3; TRAILING:3; SLIDINGWINDOW:4 : 15; MINLEN:19). Mapping of the trimmed reads to the National Center for Biotechnology Information (NCBI) virus database was performed using Bowtie2 v2.4.1 [12]. Contigs were assembled from small RNA-seq data using all trimmed reads as input for SPAdes *De Novo* Assembler [13] with the following parameter settings: only-assembler mode, coverage depth cutoff 10 and k-mer lengths of 17, 18 and 21. Assembly of contigs from RNA-seq data was performed with default settings.

Scanning of contig sequences for potential RNA-dependent RNA polymerase (RdRp)-like proteins was performed using PalmScan [14] and LucaProt [15].

## Results and discussion

### Discovery of a novel konkovirus species in *Lachenalia* plants

In our study, we employed a combination of small RNA-seq, RNA-seq, advanced bioinformatics and manual curation to uncover previously unknown plant viruses in 25 *Asparagales* plants showing mild-to-severe disease phenotypes, collected from an urban botanic garden in Amsterdam (Table ST1). The small RNA-seq experiment yielded, on average, ~11 million sequencing reads (<76 nt) per sample, and the RNA-seq experiment yielded, on average, ~39 million read pairs (2×150 nt) per sample (Table ST1).

**Fig. 1.**
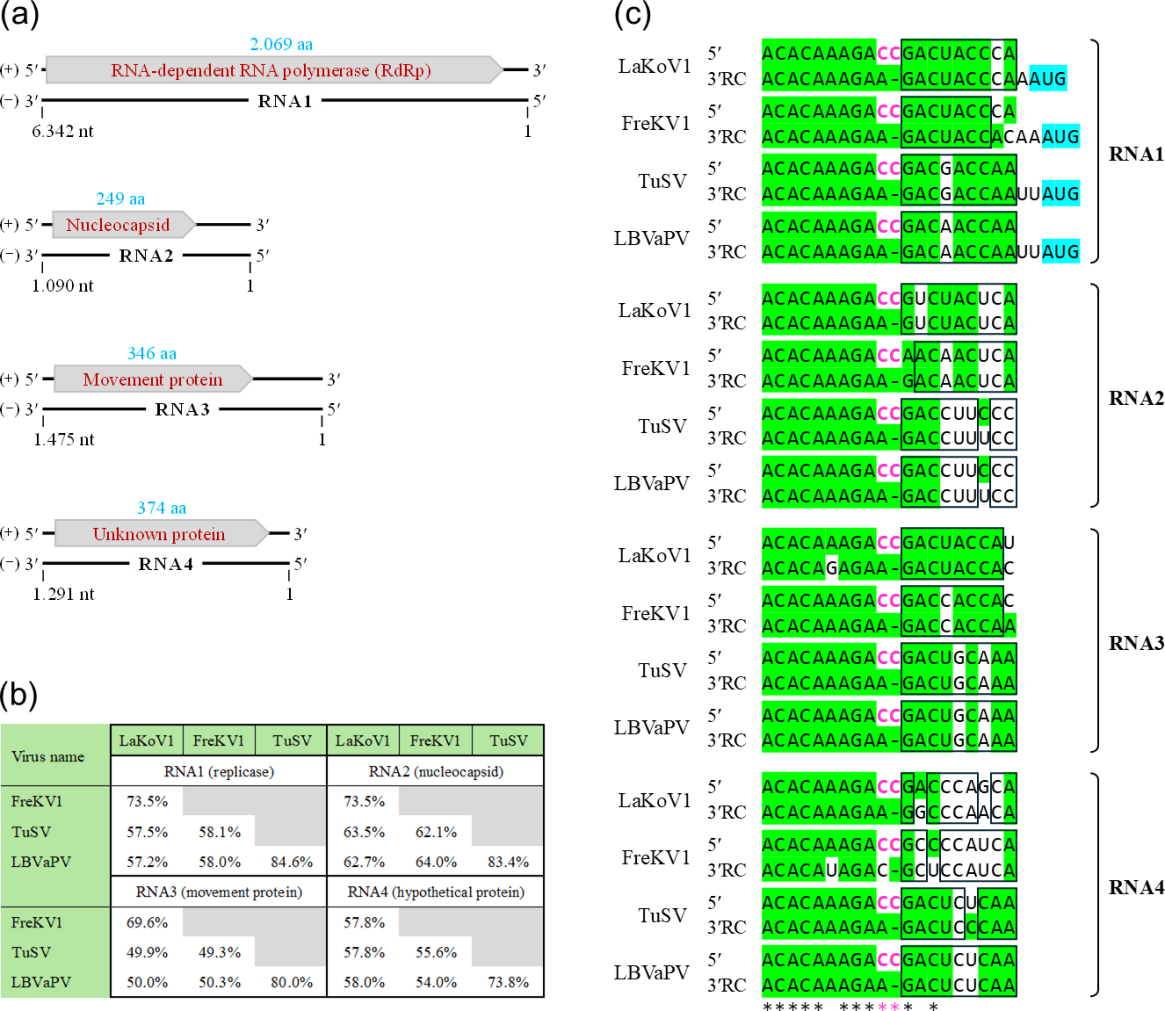
Genomic organization, sequence similarity and terminal motif features of LaKoV1. (**a**) Schematic illustration of the discovered LaKoV1 genome and its associated proteins. Indicated on the +strand are the proposed open reading frames (grey arrow box) and corresponding protein sizes (blue). (**b**)The aa similarity between (LaKoV1-NL1-26) and the three most similar konkovirus genome sequences: FreKV1, TuSV and LBVaPV. (**c**) Similarities between the 5′ and 3′ terminus motifs of the four genomic RNA segments of each virus. To facilitate interpretation of the sequence complementarity, the reverse complement of the 3′ terminal sequences (3′RC) is shown. Nucleotides identical to the consensus nucleotide are highlighted in green. The hallmark non-complementary, protruding CC at positions 10 and 11 in the 5′ motif is indicated in pink [22]. Nucleotides at positions 11 (3′) and 12 (5′) that are identical in the same RNA molecule are indicated by a black box. The AUG translation start sites for the RdRp proteins are highlighted in blue.

Initial assembly of the small RNA-seq reads resulted in several contigs that showed a weak similarity to the RdRp-containing RNA1 segment of tulip streak virus (TuSV) [16]. TuSV is a segmented, negative-sense, single-stranded RNA virus with four genome segments, classified within the genus *Olpivirus* (family *Konkoviridae*, order *Hareavirales*); it was initially reported to contain two segments of ~6 and 1.1 kb [16], but later research extended this to include two additional smaller segments of ~1.1 and 1.3 kb (Table ST2). The TuSV-like siRNA-derived contigs, combined with RNA-seq contigs, were assembled into four virus RNA segments (RNA1–RNA4; GenBank accessions no. PQ067367, PQ067368, PQ067369, PQ067370) that code for four virus-related proteins (Fig. 1a and Table ST2).

**Fig. 2.**
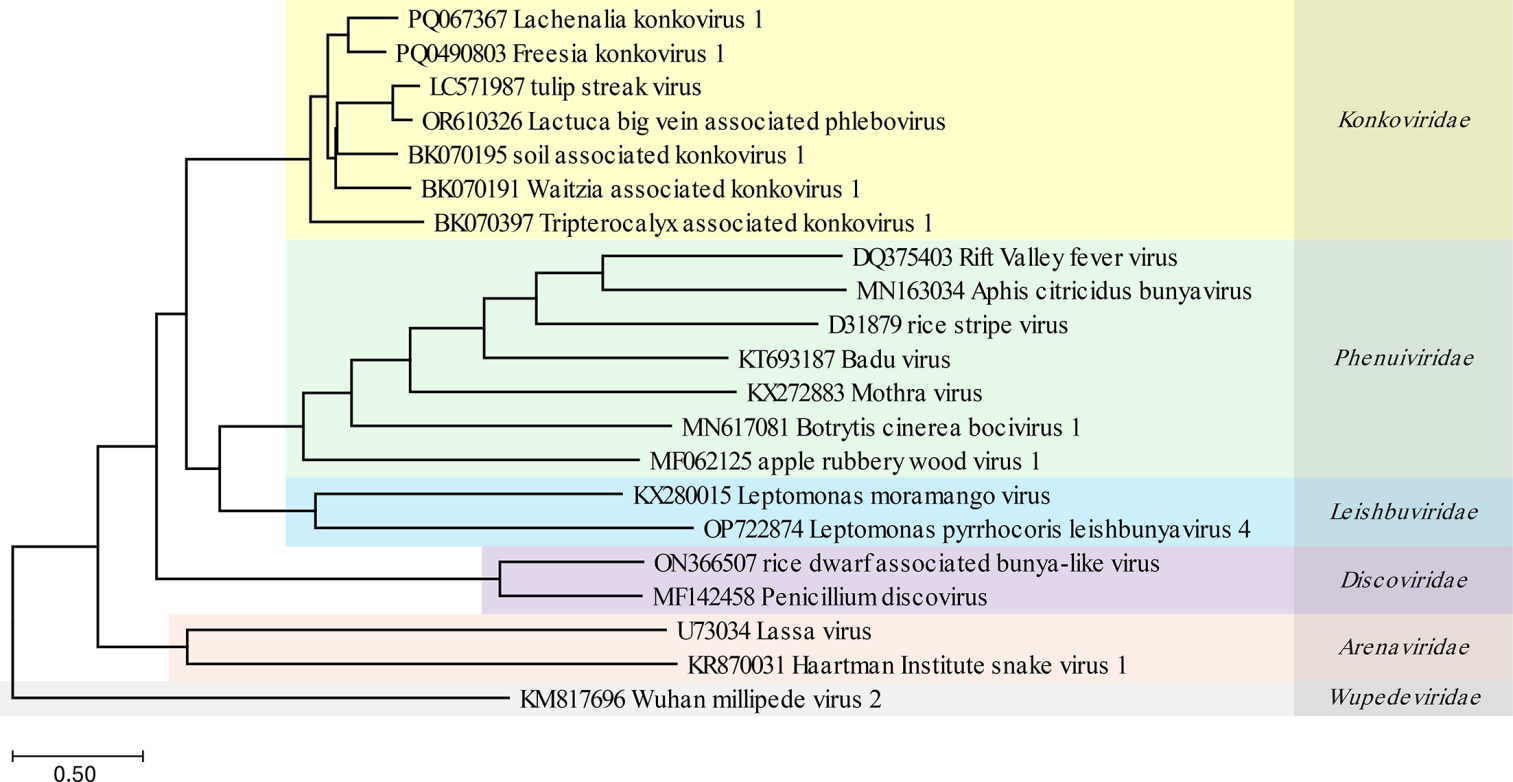
RdRp protein sequence similarity tree of LaKoV1 and representative viruses from related families. A neighbour-joining tree was constructed based on the RdRp aa sequences from LaKoV1 and representative members of related bunyavirus families. Multiple sequence alignment was performed using muscle v3.8.31 with 32 iterations. The tree was generated using the Kimura protein distance measure with 1,000 bootstrap replicates. LaKoV1 clusters within the *Konkoviridae* family (yellow shading), along with TuSV, LBVaPV and other known konkoviruses. Related viral families are colour-coded: *Phenuiviridae* (green), *Leishbuviridae* (blue), *Discoviridae* (purple), *Arenaviridae* (red) and *Wupedeviridae* (grey). The scale bar represents the estimated number of aa substitutions per site based on the Kimura protein distance model.

The similarity between the specific RNAs of this new konkovirus and the known TuSV virus ranges from 56% to 68% (with coverage of 36–72%) at the RNA level and 50% to 63% (with coverage of 87–100%) at the protein level (Fig. 1b and Table ST2). These similarities are well below the species demarcation criteria for the *Konkoviridae* family identity (<95% identity in the RdRp aa sequence) [17], supporting the conclusion that the virus sequences we found represent a previously unknown konkovirus species. Consistently, when visualized in an RdRp protein sequence similarity tree that includes representatives from the most closely related viral families, the novel virus clusters within the *Konkoviridae* clade, further supporting its classification as a novel member of this family (Fig. 2). The *Konkoviridae* is a relatively new family, with, at the time of publication, only two officially recognized species [TuSV and *Lactuca* big vein-associated *Phlebovirus* (LBVaPV)]. Additional konkovirus genomes [*Freesia konkovirus* 1 (FreKV1), soil associated konkovirus (SaKV), Waitzia associated konkovirus 1 (WaKV1), and Tripterocalyx associated konkovirus 1 (TaKV1)] are available, but formal classificiation by the International Committee on Taxonomy of Viruses (ICTV) is pending.

When we mapped the small RNA-seq and RNA-seq reads from all samples back to the new virus RNA sequences, we found evidence for siRNA related to this virus in four samples (S09, S15, S22 and S26), all of which were *Lachenalia* samples (Tables ST1 and ST3). Hence, we named our new virus *Lachenalia konkovirus* 1 (LaKoV1), and the isolation variant sequence from sample 26 (LaKoV1-NL1-26) was used in our subsequent analyses.

One additional konkovirus sequence in GenBank, LBVaPV, also showed similarity to both LaKoV1 and TuSV [18] (Table ST2). Even though TuSV and LBVaPV differ substantially, with a similarity of 69–75% at the RNA level and 74–85% at the protein level, they show almost the same weak similarity to LaKoV1, which results in a triangular sequence distance from each other (Fig. 1b and Table ST2). This is clear at the protein level, where the similarity coverage is always at least 87%, whereas at the RNA level, this coverage goes even as low as 22%. The fact that the overall RNA similarity is lower than protein similarity is a common occurrence in viral genome comparisons. Given that these three distinct viruses were found in plants from three different orders, *Asparagales* (LaKoV1), *Liliales* (TuSV) and *Asterales* (LBVaPV), this might explain the observed genomic distances.

**Fig. 3.**
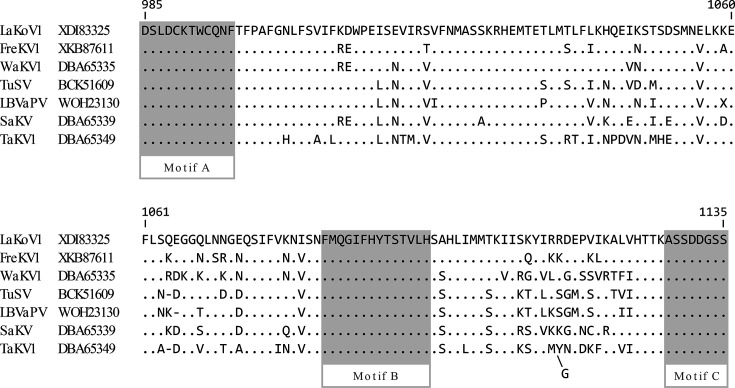
Sequence comparison of the conserved RdRp palm subdomains with the characteristic motifs. Alignment of the LaKoV1 conserved RdRp palm subdomain protein sequences with the three most similar RdRp subdomain sequences in GenBank. The aa sequences of the RdRp subdomain motifs A, B and C are shaded in grey. The aa sequences that diverge from the LaKoV1 RdRp subdomain motifs are highlighted in red.

When we zoomed in on the RdRp subdomain region with the well-conserved motifs A, B and C, which play an essential role in catalytic function of RdRp [19], it was clear that while there was substantial difference between LaKoV1 and TuSV (19%) or LBVaPV (19%), the three sequences of the three domains were identical (Fig. 3), even though they represent 23% of the subdomain sequences. The next most similar RdRp subdomain (*Arenavirus* from the *Arenaviridae* family) contained divergent aa sequences in each of the three domains (Fig. 3), suggesting that although the LaKoV1, TuSV and LBVaPV are different species, they are evidently related.

We also observed a noticeable difference in relative small RNA-seq read counts between the RNA segments of this new virus. After correction for read length, the relative read counts for the RNA1 and RNA4 segments were 0.11 and 0.34 times the overall average read count, whereas for RNA2 and RNA3 segments, these values were 2.31 and 1.24, respectively (Table ST4). This difference in small RNA read counts was consistent across all four LaKoV1-positive samples. As most of those reads are 21-mers, this means that, for unknown reasons, the relative number of siRNAs for each RNA fragment is rather dissimilar. This phenomenon is also present with RNA-seq reads, with low relative read counts (RNA1, 0.22; RNA3, 0.78) and high relative read counts (RNA2, 1.91; RNA4, 2.64). RNA-seq reads represent either virus RNA or virus mRNA. Hence, there is no direct correlation detectable between the relative number of siRNAs and virus (m)RNA (Table ST4).

The four RNA segments of the LaKoV1 virus contain complementary 5′ and 3′ terminal sequences that can form a so-called ‘panhandle structure’, which is typical for bunyaviruses [17,20, 21]. When comparing these terminal sequences with those of other *Konkoviridae*, we find a nine-nucleotide stretch that is completely conserved in all but one terminal sequence of FreKV1, TuSV and LBVaPV (Fig. 1c). In contrast, the other *Konkoviridae* (TaKV1, SaKV and WaKV1) do not show clear conservation, which may be due to incomplete genome ends in the available sequence data (Table ST5). The 5′ sequence continues with a conserved CC, whereas the 3′ sequence shows one T, leading to an important protruding C nucleotide when both ends pair [22]. Comparing the sequence termini of these konkoviruses with other four- or eight-segmented phenuiviruses (Fig. S1) revealed, next to the hallmark non-complementary CC nucleotides at nucleotide positions 10 and 11 in the 5′ terminus motif, a possible importance for positions 9 (5′ and 3′) and 10 (3′). These nucleotides invariably are (for the 3′ sequence in reverse complement orientation) UC in tenuiviruses and mechloroviruses, AC in wenriviruses (Fig. S1) and AA in the three somewhat similar konkoviruses in this study, of which the previously reported viruses are linked to *Phleboviruses*, even though this genus reportedly contains only three segments [17].

In LaKoV1, FreKV1, TuSV and LBVaPV, the second stretch of nine nucleotides in the terminal sequences seems to be more virus/RNA-segment specific, as no terminal sequence occurs in more than one RNA segment for all three viruses. Moreover, compared to the consensus sequence of this second nucleotide stretch, there are 33 non-consensus nucleotide pairs (31%) of the total 108 pairs. Yet more striking, only six nucleotide pairs (5.6%) are non-complementary. Thus, even though there are quite some differences in the second stretch, there are few non-complementary divergences, which might be extremely important for vital virus functions, as shown by Kohl *et al*. [23] through the effects of a single-point mutation in the 3′ terminus of a bunyavirus. The inclination towards maintaining complementarity might indicate a form of co-evolution between the 5′ and 3′ termini of the RNA segment in these konkoviruses.

## Concluding remarks

With this study, we add a new virus species to the ever-expanding global virome. In our research on virus presence in (urban) botanic gardens, this is the second novel virus species we report [10]. The first novel virus species was a single-stranded, positive-sense RNA virus belonging to the genus *Capillovirus* (family *Betaflexiviridae*). Here, we describe a single-stranded, negative-sense RNA virus classified within the family *Konkoviridae*.

The observation that the siRNA response to the four virus RNA segments, as well as the presence of these virus RNA molecules, is both different and not related, yet quite consistent between samples, is puzzling. Constant variation in the presence of viral RNA or viral mRNA is explicable; however, constant variation in virus-related siRNA hints at an siRNA regulatory mechanism based on the sequences of the RNA segments. Most likely, this is a cumulative effect of siRNA hot spots and cold spots in the various RNA segments [24].

With our new konkovirus, we detected an indication of a possible presence of sequence co-evolution of the 5′ and 3′ termini that can form so-called panhandle RNA structures in the four molecules, probably involved in RNA replication, transcription, pseudo-circularization and virus packaging [21,22, 25,28]. Each addition of well-analysed and well-annotated virus sequences to virus databases increases the potential for new discoveries in the mechanisms of viruses, which may be beneficial to the battles against pathogenic viruses.

## Supplementary material

10.1099/jgv.0.002159 Uncited Supplementary Material 1.

## Abbreviations

FreKV1: Freesia konkovirus 1
ICTV: International Committee on Taxonomy of Viruses
LaKoV1: Lachenalia konkovirus 1
LBVaPV: *Lactuca* big vein-associated *Phlebovirus*
NCBI: National Center for Biotechnology Information
RdRp: RNA-dependent RNA polymerase
RNA-seq: RNA sequencing
SaKV: soil associated konkovirus
siRNA: small interfering RNA
TaKV1: Tripterocalyx associated konkovirus 1
TuSV: tulip streak virus
WaKV1: Waitzia associated konkovirus 1
